# Right to left ventricular volume ratio is associated with mortality in congenital diaphragmatic hernia

**DOI:** 10.1038/s41390-022-02430-z

**Published:** 2023-01-09

**Authors:** Katsuaki Toyoshima, Tomoko Saito, Tomoyuki Shimokaze, Kaoru Katsumata, Junya Ohmura, Sasagu Kimura, Hirosato Aoki, Megumi Takahashi, Jun Shibasaki, Motoyoshi Kawataki, Ki-Sung Kim, Masato Shinkai, Hiroshi Ishikawa, Naka Saito, Satoshi Masutani

**Affiliations:** 1 Department of Neonatology, Kanagawa Children’s Medical Center, Yokohama, Japan; 2 Department of Cardiology, Kanagawa Children’s Medical Center, Yokohama, Japan; 3 Department of Surgery, Kanagawa Children’s Medical Center, Yokohama, Japan; 4 Department of Obstetrics, Kanagawa Children’s Medical Center, Yokohama, Japan; 5 Department of Clinical Laboratory, Kanagawa Children’s Medical Center, Yokohama, Japan; 6 Department of Pediatrics, Saitama Medical University, Kawagoe, Japan

## Abstract

**Background:**

Congenital diaphragmatic hernia (CDH) is associated with high neonatal mortality. We performed this study to test the hypothesis that left ventricular (LV) and right ventricular (RV) volumes assessed by three-dimensional echocardiography may be associated with mortality in CDH.

**Methods:**

This study was a single-center retrospective cohort study involving 35 infants with CDH. RV and LV end-diastolic volume (RVEDV and LVEDV, respectively) were measured by three-dimensional echocardiography and were corrected by birth body weight (BBW) on day 1. RVEDV/BBW, LVEDV/BBW, and LVEDV/RVEDV were compared between CDH survivors and non-survivors. Receiver-operating characteristic curve analysis was performed to assess the predictive ability for mortality of the echocardiographic parameters.

**Results:**

Comparing CDH non-survivors (*n* = 6) with survivors (*n* = 29), respectively, RVEDV/BBW was significantly larger (2.54 ± 0.33 vs 1.86 ± 0.35 ml/kg; *P* < 0.01), LVEDV/BBW was significantly smaller (0.86 ± 0.21 vs 1.22 ± 0.33 ml/kg; *P* < 0.001), and LVEDV/RVEDV was significantly lower (0.34 ± 0.06 vs 0.66 ± 0.18; *P* < 0.001). The area under the curve for LVEDV/RVEDV was the largest (0.98).

**Conclusions:**

Three-dimensional echocardiographic volume imbalance between the RV and LV was remarkable in CDH non-survivors. The LVEDV/RVEDV ratio may be associated with mortality in CDH.

**Impact:**

- Mortality with congenital diaphragmatic hernia (CDH) is high, and evaluating left and right ventricular structures and functions may be helpful in assessing the prognosis.
- Three-dimensional (3D) echocardiography indicated that the left ventricular end-diastolic volume/right ventricular end-diastolic volume ratio within 24 h after birth was associated with mortality in CDH infants.
- The usefulness of this ratio should be validated in prospective multicenter studies involving larger numbers of patients.

## Introduction

Congenital diaphragmatic hernia (CDH) is characterized by herniation of the abdominal contents into the thorax. Despite advances in neonatal intensive care and surgical management, CDH continues to cause significant mortality and morbidity.^1,2^ Pulmonary hypoplasia and persistent pulmonary hypertension of the newborn (PPHN) because of abnormal pulmonary vascular development play central roles in CDH pathophysiology,^3–5^ with increased right ventricular (RV) afterload and volume. There is also increasing recognition that left ventricular (LV) function may be another key determinant of CDH severity.^6–10^ Limited LV dilation capacity enhanced by compression by an enlarged RV and decreased pulmonary venous return owing to PPHN synergistically reduce LV volume and output. Because ventricular volume is determined by loading conditions and ventricular stiffness, and reflects fetal organogenesis and hemodynamics, RV and LV volumes and their balance may reflect the severity in CDH.

Evaluating the volumes of the deformed RV and LV in CDH is difficult using traditional two-dimensional echocardiography because the RV has a complicated shape, and the assumption that the LV is spheroidal does not hold in neonatal CDH. Cardiac magnetic resonance (CMR) imaging, the gold standard measurement of ventricular volume in children and adults, carries a life-threatening risk in CDH, where even moving to the imaging room may exacerbate PPHN. In contrast, three-dimensional (3D) echocardiography can be used at the bedside to determine cardiac chamber volume, independent of angle and without making geometric assumptions,^11,12^ with high accuracy and reproducibility, similar to those with CMR.^13–18^ Previous studies reported that 3D echocardiography can assess LV and RV volume in newborn infants.^19,20^ Accordingly, this study was undertaken to test the hypothesis that LV and RV volumes assessed by 3D echocardiography within the first 24 h after birth are associated with mortality in infants with CDH.

## Methods

### Study design and population

This study was a single-center retrospective cohort study that involved infants with CDH with gestational age ≥36 weeks who were admitted to the neonatal intensive care unit (NICU) within 24 h after birth between January 2017 and June 2021. Infants with significant congenital anomalies, such as cardiac anomalies other than patent foramen ovale, patent ductus arteriosus, and persistent left superior vena cava, as well as multiple abnormalities, were excluded. Because of the paucity of 3D echocardiography data in this population, a power calculation was not performed.

Newborn infants with prenatally diagnosed CDH underwent routine fetal ultrasonography examination. The severity of the condition was quantified by measuring the observed/expected lung-to-head ratio (o/e LHR) and the lung/thorax transverse area ratio during the fetal period.^21,22^ CDH cases were managed by a multidisciplinary team in accordance with an institutional treatment protocol, in accordance with Japanese clinical guidelines.^23^

### Clinical characteristics

Data on sex, gestational age, birth weight, Apgar scores, and mode of delivery were collected from the electronic medical records. In the CDH group, additional characteristics, side of the defect, treatment, and outcome data were obtained, namely age at repair, stomach and liver positions, cardiovascular therapies, days of age at repair, and survival to neonatal discharge.

The respiratory severity score (RSS) at the time of initial echocardiography was calculated as mean airway pressure (mmHg) × fraction of inspired oxygen.^24^ The vasoactive–inotropic score (VIS) at the initial echocardiography was calculated as dopamine dose (mg/kg/min) + dobutamine dose (mg/kg/min) + 100 × epinephrine dose (mg/kg/min) + 100 × norepinephrine dose (mg/kg/min) + 10,000 × vasopressin dose (U/kg/min) + 10 × milrinone dose (mg/kg/min).^25^ The oxygenation index (OI) at the time of initial echocardiography was calculated using the following formula: (% inspired oxygen × mean airway pressure (mmHg) × 100)/arterial partial pressure of oxygen (mmHg).

### Echocardiographic measurements

Echocardiographic data were collected by a single experienced echocardiographer (K Toyoshima).RV and LV volumes were assessed using 3D echocardiography as part of our institutional protocol. Full-volume 3D echocardiography datasets were acquired by the apical approach using a commercially available ultrasound machine and equipment (EPIC 7 G or EPIC CVx with the X7-2 probe; Philips Healthcare, Andover, MA). The depth and sector angle were adjusted to include the entire RV or LV with the frame rate of >40 frames per second. To encompass the complete RV or LV into the 3D dataset, a full-volume scan was acquired from six R wave-triggered subvolumes. There were six cardiac cycles per capture, which were stitched together. We extracted 3D data from captures of six cardiac cycles with deep sedation, no body motion, and no change in loading conditions.

The 3D echocardiography analysis for LV and RV volume measurements was performed by an experienced investigator (K Toyoshima) using a novel 3D echocardiography software (4D LV-Analysis version 3, 4D RV-Function version 3; TomTec Imaging Systems, Unterschleissheim, Germany) for which the accuracy and reproducibility have been extensively validated by comparison with CMR.^26–28^

To obtain the LV volume, the LV endocardial border in the LV-focused four-chamber view was semi-automatically determined after two-point clicking of the LV apex and the center of the mitral valve annulus on the apical four‐chamber, two-chamber, and long‐axis views extracted from 3D echocardiography datasets. The endocardial border was manually adjusted, when required. The software generated time domain LV volume curves, from which LV volumes and LV ejection fraction (LVEF) were calculated (Supplementary Fig. 1). LV end-diastolic volume (LVEDV), end-systolic volume (LVESV), stroke volume (LVSV), LVEF, global longitudinal strain (LVGLS), global circumferential strain (LVGCS) and torsion (LV torsion) were automatically generated by the software (Supplementary Fig. 1).

**Fig. 1:**
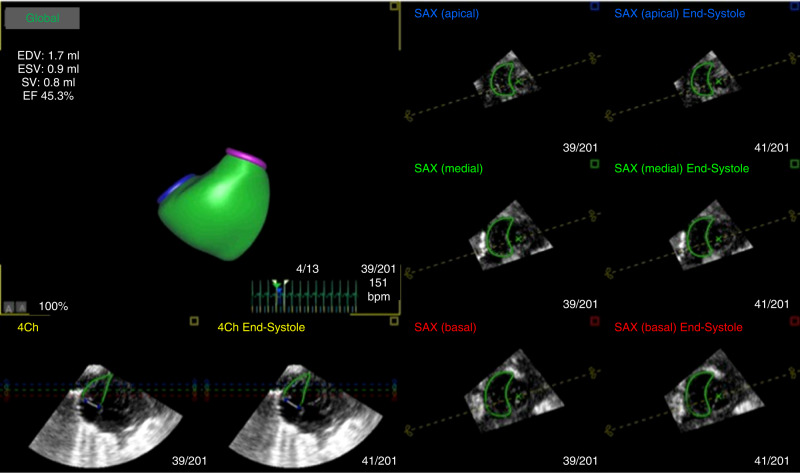
Offline analysis of three-dimensional echocardiographic right ventricular (RV) volume calculations at end-diastole. Green lines indicate semi-automatically detected RV internal border. EDV end-diastolic volume, ESV end-systolic volume, SV stroke volume, EF ejection fraction, SAX short axis, 4Ch four chamber.

To obtain RV volume, three orthogonal planes and various landmarks in the apical RV-focused four-chamber view were selected to define the ED frames (Fig. 1). According to the initial view adjustment, the program automatically supplied four-chamber, sagittal, and coronal RV views as well as RVED volume (RVEDV), RVES volume (RVESV), RV stroke volume (RVSV), RVEF, RV longitudinal free-wall (RVLS free-wall) and RV septal strain (RVLS septum) (Fig. 1). We then calculated the LVEDV/RVEDV ratio to assess the balance between the two ventricles. In addition, the left ventricular end-diastolic diameter and EF were calculated with the M-mode images from the long axis view. The right and left ventricular diameter (RVD and LVD) at end-diastole were measured in the apical four-chamber (A4C) view with a focus on the right ventricle.^29^ We then calculated the LVD/RVD ratio in the A4C view to assess the balance between the two ventricles.

We calculated left atrial (LA) volume using the single-plane area–length method with the following equation: LA volume|=|0.85|×|(LA area)^2^/(LA length) (cm^3^) in the four-chamber view.^30^ Body size-dependent parameters were divided by the infant’s birth body weight (BBW).

### Reproducibility analyses

To investigate intraobserver variability, 15 studies were randomly selected from the CDH group, with one observer (K Toyoshima) measuring RV and LV volume at 6-month intervals. The observer was blinded to the results of the first measurement. A second observer (H Aoki) independently analyzed these data and was blinded to the results of the first observer. Intra- and inter-observer variabilities were examined by intra-class correlation coefficient (ICC) and Bland–Altman analysis.

### Statistical analyses

Descriptive statistics (e.g., mean ± standard deviation, median [interquartile range]) were used to summarize the demographic or clinical data of the infants in CDH survivors and non-survivors. Differences between two groups were analyzed using the unpaired *t* test for continuous variables, Mann–Whitney U-test for median values, or Fisher’s exact test for categorical data.

We used univariate logistic regression to predict mortality and calculated the estimated areas under the receiver operating characteristic curves (area under the curve (AUC)) of the models. The optimal cut-off value was determined as the maximum Youden index. We described AUC values of 0.9–1.0, 0.8–0.9, 0.7–0.8, 0.6–0.7, and 0.5–0.6 as excellent, good, fair, poor, and fail, respectively.^31,32^ Statistical analyses were performed with EZR (version 1.54) (Saitama Medical Center, Jichi Medical University, Saitama, Japan), which is a graphical user interface for R (The R Foundation for Statistical Computing, Vienna, Austria) and MedCalc (version 20; MedCalc Software Ltd., Ostend, Belgium). A *P* value of <0.05 was considered significant. This study was conducted in accordance with the principles contained in the Declaration of Helsinki and was approved by the institutional review board of Kanagawa Children’s Medical Center (No. 2011–14; March 2021).

## Results

### Clinical data

**Fig. 2:**
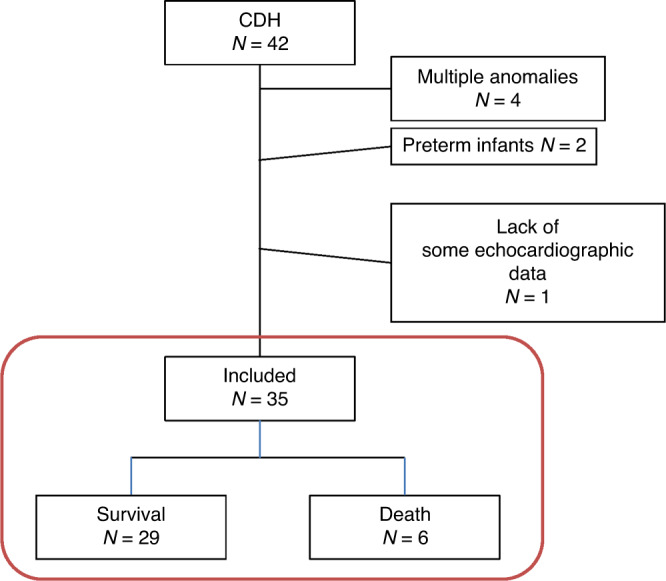
Study flow chart. A total of 42 infants with CDH were admitted to our hospital. Seven patients were excluded owing to the presence of multiple anomalies (*n* = 4), preterm birth (*n* = 2), or the lack of some echocardiographic data (*n* = 1). Thirty-five patients were included in the final analysis, of whom 29 survived (83%). CDH congenital diaphragmatic hernia.

Between January 2017 and June 2021, among 42 infants with CDH who were admitted to our hospital, 35 were included in the analyses (Fig. 2). Among the 35 CDH infants, 30 infants (85.7%) underwent surgical repair at a median (range) of 4 (3–10) days of age. Thirty patients (85.7%) were ventilated using high-frequency oscillation (HFO), while five cases that did not have significant respiratory distress, cyanosis, or hypercapnia were ventilated by conventional mechanical ventilation (CMV). All 35 CDH infants (100%) received cardiotropes (dopamine and dobutamine), and 31 infants (88.6%) received inhaled nitric oxide at the time of the echocardiography. During echocardiography, the non-survivor group received slightly more intravenous fluids compared with the survivor group (75 (68–80) vs 70 (66–73) mL/kg/day; *P* = 0.031; Table 1). No infant received prostaglandin E_1_ infusion at echocardiography. No infant received extracorporeal membrane oxygenation.

**Table 1:** Demographic and CDH characteristics, and treatment, outcomes, hemodynamic, and echocardiographic data within 24 h after birth in CDH survivors and CDH non-survivors.

	CDH survivors	CDH non-survivors	*p* value
Number of patients	29	6	
Gestational age (weeks)	38.8 ± 1.2	38.3 ± 1.5	0.39
Birth weight (g)	2897 ± 475	2820 ± 240	0.70
Male sex, n (%)	16 (55.2)	6 (100%)	0.064
In-hospital birth, n (%)	25 (86.2)	6 (100%)	1.0
Cesarean section, n (%)	7 (24.1)	3 (50%)	0.32
Apgar score, 1 min, median (range)	5 (1–9)	2.5 (1–4)	0.001
Apgar score, 5 min, median (range)	8 (3– 9)	5.5 (4–7)	<0.001
Side of defect (right), n (%)	4 (13.7%)	2 (33.3%)	0.26
Liver up, n (%)	9 (31.0%)	6 (100%)	0.003
Stomach up, n (%)	11 (37.9%)	5 (83.3%)	0.072
o/e LHR	54.7 ± 21.3	21.8 ± 12.9	0.001
L/T ratio	0.14 ± 0.04	0.05 ± 0.01	<0.001
HFO, n (%)	24 (82.7%)	6 (100%)	0.56
iNO, n (%)	25 (86.2%)	6 (100%)	1.0
Operation, n (%)	29 (100%)	1 (16.7%)	<.001
ECMO, n (%)	0 (0%)	0 (0%)	1.0
At echocardiography			
Hours after birth (h)	6.5 (4.5–12)	3.5 (3.1–6.1)	0.079
Fluid volume (ml/kg/day)	70 (66–73)	75 (68–80)	0.031
Mean airway pressure (MAP) (mmHg)	13 (12–14)	14 (12–16)	0.062
Respiratory severity score (RSS)	11.2 (8.4–14)	14 (14–14.8)	0.011
Vasoactive–inotropic score (VIS)	6.0 (6.0–8.0)	10.5 (10.0–11.8)	0.008
Oxygenation index (OI)	4.0 (3.3–5.9)	54.9 (42.9–77.6)	<0.001
AaDO_2_ (mmHg)	262 (160–396)	615 (562–632)	0.003
Systolic blood pressure (mmHg)	58.3 ± 7.6	52.8 ± 11,6	0.151
Diastolic blood pressure (mmHg)	39.7 ± 5.9	36.0 ± 7.6	0.196
Heart rate (bpm)	130 ± 18	142 ± 21	0.144
2D echocardiography			
LVDD (M mode, 2D) (mm)	14.8 ± 6.9	12.3 ± 2.6	0.057
LVDD/BBW (M mode, 2D) (mm)	5.16 ± 0.80	3.55 ± 1.9	0.002
LVEF (M mode, 2D) (%)	77.6 ± 9.6	64.4 ± 15.7	0.028
LAVI (ml/kg)	0.44 ± 0.19	0.18 ± 0.09	0.006
LVD (RV focused A4CV) (mm)	11.1 ± 1.8	10.0 ± 1.8	0.20
RVD (RV focused A4CV) (mm)	14.7 ± 2.4	17.6 ± 2.5	0.01
LVD/RVD (RV focused A4CV)	0.77 ± 0.15	0.57 ± 0.06	<0.001
Patent ductus arteriosus			
Closed, n (%)	1 (3.5%)	0 (0%)	1
Right to left flow, n (%)	2 (6.9%)	1 (16.7%)	0.44
Bidirectional, n (%)	19 (65.6%)	5 (83.3%)	0.30
Left to right flow, n (%)	7 (24.1%)	0 (0%)	0.31
3D echocardiography			
RVEDV (ml)	5.39 ± 1.25	7.17 ± 1.11	0.003
RVEDV/BBW (ml/kg)	1.86 ± 0.35	2.54 ± 0.33	<0.001
RVESV (ml)	3.18 ± 0.95	4.90 ± 0.83	<0.001
RVESV/BBW (ml/kg)	1.10 ± 0.28	1.17 ± 0.25	<0.001
RVSV (ml)	2.10 ± 0.78	2.27 ± 0.85	0.64
RVSV/BBW (ml/kg)	0.73 ± 0.26	0.80 ± 0.31	0.52
RVEF (%)	38.5 ± 9.2	31.3 ± 9.5	0.091
RVCO (ml/kg/min)	94 ± 34	116 ± 56	0.20
RVLS (FW) (%)	−19.1 ± 7.3	−14.1 ± 7.2	0.13
RVLS (S) (%)	−19.6 ± 7.5	−15.6 ± 6.5	0.23
LVEDV (ml)	3.53 ± 1.10	2.40 ± 0.51	0.020
LVEDV/BBW (ml/kg)	1.22 ± 0.33	0.86 ± 0.21	0.015
LVESV (ml)	1.77 ± 0.61	1.38 ± 0.21	0.012
LVESV/BBW (ml/kg)	0.61 ± 0.20	0.50 ± 0.10	0.164
LVSV (ml)	1.76 ± 0.74	0.98 ± 0.31	0.018
LVSV/BBW (ml/kg)	0.60 ± 0.23	0.35 ± 0.12	0.016
LVEF (%)	49.0 ± 11.8	40.3 ± 7.4	0.095
LVCO (ml/kg/min)	76 ± 28	49 ± 19	0.033
LVGCS (%)	−21.7 ± 6.3	−16.4 ± 2.8	0.056
LVGLS (%)	−15.0 ± 5.9	−10.5 ± 4.4	0.087
LV torsion (°/cm)	4.0 ± 2.7	3.7 ± 2.4	0.83

Data are presented as mean ± standard deviation, median (interquartile range) or number (percent). Student’s *t* test and Fisher’s exact test were used for continuous and categorical variables, respectively.

Differences in median values between the groups were compared using the Mann–Whitney U-test.

*P < 0.05 for comparisons between the CDH survivors and non-survivors.

*CDH* congenital diaphragmatic hernia, *o/e LHR* observed/expected lung-to-head ratio, *L/T ratio* lung/thorax transverse area ratio, *HFO* high- frequency oscillation, *iNO* inhaled nitric oxide, *ECMO* extracorporeal membrane oxygenation, *AaDO*_*2*_ alveolar–arterial oxygen difference, *2D* two-dimensional, *3D* three-dimensional, *LVDD* left ventricular diastolic dimension, *LVEF* left ventricular ejection fraction, *LAVI* left atrial volume index, *LVD* left ventricular diameter, *RVD* left ventricular diameter, *A4CV* apical four chamber view, *RVEDV* right ventricular end-diastolic volume, *RVESV* right ventricular end-systolic volume, *RVSV* right ventricular stroke volume, *RVEF* right ventricular ejection fraction, *RVCO* right ventricular cardiac output, *RVLS* right ventricular longitudinal strain, *FW* free wall, *S* interventricular septum, *LVEDV* left ventricular end-diastolic volume, *LVESV* left ventricular end-systolic volume, *LVSV* left ventricular stroke volume, *LVCO* left ventricular cardiac output, *LVGCS* left ventricular global circumferential strain, *LVGLS* left ventricular global longitudinal strain, *LV torsion* left ventricular torsion, *BBW* birth body weight.

Six infants in the CDH group (17.1%) died at a median (range) of 2 (1–42) days of age. Five of the six infants without CDH surgery died 25 (7–34) hours after birth because of marked hypoxia and deterioration despite intensive care (Table 1). The parents of these children elected palliative care, and we did not pursue extracorporeal membrane oxygenation or CDH surgery. Echocardiography was not used as part of this decision-making process. Six of the 35 cases were right-sided CDH. Four of 29 infants in the survivor group and 2 of 6 in the non-survivor group had right-sided CDH. There was no significant difference in the side of CDH between the survivor and non-survivor groups.

### Reproducibility of 3D measurements

**Fig. 3:**
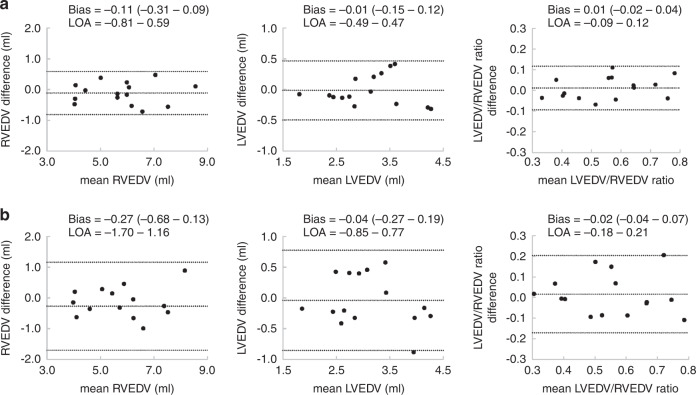
Intra- and inter-observer variabilities. Intra- (**A**) and inter-observer (**B**) variabilities assessment. The three dashed lines show biases (means of differences) and LOA. Bias is expressed as the mean of the difference (95% CI). LOA is shown as bias ± 2 SD. LOA limits of agreement, RVEDV right ventricular end-diastolic volume, LVEDV left ventricular end-diastolic volume, SD standard deviation, CI confidence interval.

The intra- and interobserver variabilities analysis in newborn infants with CDH, including the percentage bias, 95% limits of agreements, and ICCs for RVEDV, LVEDV, and the RVEDV/LVEDV ratio are summarized in Table 2. Intra- and inter-rater reproducibility were excellent (ICCs for RVEDV, LVEDV, and LV/RV ratios: 0.98, 0.97, 0.96, and 0.92, 0.92, 0.89, respectively). Bland–Altman plots are shown in Fig. 3. The bias values were not significant, and the limits of agreement were acceptable.

**Table 2:** Intra- and inter-observer reproducibilities.

Intra-observer reproducibility			
	Bland–Altman analysis		ICC (95% CI)
	Bias (95% CI)	LOA	
RVEDV	−0.11 (−0.31 – 0.09)	−0.81 – 0.59	0.98 (0.95 – 0.99)
LVEDV	−0.01 (−0.15 – 0.12)	−0.49 – 0.47	0.97 (0.91 – 0.99)
LVEDV/RVEDV ratio	0.01 (−0.02 – 0.04)	−0.09 – 0.12	0.96 (0.89 – 0.99)

Inter-observer reproducibility			
	Bland–Altman analysis		ICC (95% CI)
	Bias (95% CI)	LOA	
RVEDV	−0.27 (−0.68 – 0.13)	−1.70 – 1.16	0.92 (0.77 – 0.97)
LVEDV	−0.04 (−0.27 – 0.19)	−0.85 – 0.77	0.92 (0.76 – 0.97)
LVEDV/RVEDV ratio	0.02 (−0.04 – 0.07)	−0.18 – 0.21	0.89 (0.67 – 0.96)

*RVEDV* right ventricular end-diastolic volume, *LVEDV* left ventricular end-diastolic volume, *LV/RV ratio* left ventricular end-diastolic volume/right ventricular end-diastolic volume ratio, *CI* confidence interval, *LOA* limit of agreement, *ICC* intraclass correlation coefficient.

ICC estimates and their 95% confidence intervals were calculated using MedCalc® Statistical Software, version 20 (MedCalc Software, Ltd., Ostend, Belgium) based on a mean-rating (k = 2), absolute-agreement, two-way mixed-effects model.

### Echocardiographic findings

Echocardiography was performed in all patients within 24 h after birth. Thirty-four of the 35 included patients (97%) had patent ductus arteriosus at the time of echocardiography. Among the included patients, 19 (54.3%) had a bidirectional shunt, 7 (20%) had a left-to-right dominant shunt, and 3 (8.6%) had a right-to-left dominant shunt. There were no significant differences in the ductal closing or the direction of ductal flow between the survivor and the non-survivor groups (Table 1).

Right ventricular function in infants with CDH on day 1 measured by 3D echocardiography was as follows: RVEDV/BBW (ml/kg): 1.98 ± 0.43, RVESV/BBW (ml/kg): 1.21 ± 0.37, RVSV/BBW (ml/kg): 0.74 ± 0.27, RVEF (%): 37.3 ± 9.6, and RV cardiac output (ml/kg/min): 98 ± 39. Left ventricular function in infants with CDH on day 1 measured by 3D echocardiography was as follows: LVEDV/BBW (ml/kg): 1.16 ± 0.34, LVESV/BBW (ml/kg): 0.59 ± 0.19, LVSV/BBW (ml/kg): 0.56 ± 0.24, LVEF (%): 47.5 ± 11.6, and LV cardiac output (ml/kg/min): 72 ± 29. The LVEDV/RVEDV ratio was 0.60 ± 0.21.

### Comparison between CDH survivors and CDH non-survivors

Table 1 summarizes the comparisons between the 29 CDH survivors and 6 CDH non-survivors. Compared with CDH survivors, CDH non-survivors had lower Apgar scores, and smaller lung/thorax transverse area ratio and o/e LHR during the fetal period, indicating more severe lung hypoplasia in CDH non-survivors. CDH non-survivors had higher respiratory severity scores, and higher VIS, OI, and alveolar-arterial oxygen pressure difference values at echocardiography than those for the CDH survivors, indicating much more severe conditions with an even stronger level of intensive care in the CDH non-survivors.

**Fig. 4:**
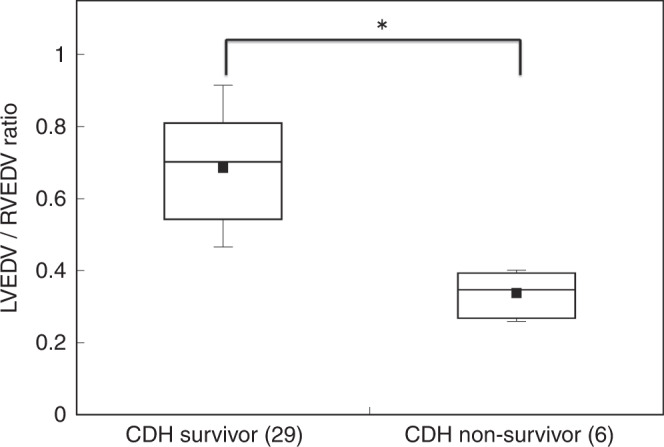
Comparison of the LVEDV/RVEDV ratios between CDH survivors and CDH non-survivors. Compared with CDH survivors, CDH non-survivors had a smaller LVEDV/RVEDV ratio. Each box plot represents the median (thick line), average (black square), and interquartile range (edges of the box). The whiskers illustrate the range of the values. *p < 0.05 between the groups. LVEDV left ventricular end-diastolic volume, RVEDV right ventricular end-diastolic volume, CDH congenital diaphragmatic hernia.

Compared with the CDH survivors, the CDH non-survivors had significantly larger RVEDV/BBW (2.54 ± 0.33 vs 1.86 ± 0.35 ml/kg; *P* < 0.001), and smaller LVEDV/BBW (0.86 ± 0.21 vs 1.22 ± 0.33 ml/kg; *P* < 0.01) and LVEDV/RVEDV ratio (0.34 ± 0.06 vs 0.66 ± 0.18; *P* < 0.001) (Fig. 4), respectively. As shown in Table 1, significant differences between CDH survivors and CDH non-survivors were not observed for RV EF and RV cardiac output. In contrast, LVEF tended to be smaller, and LV cardiac output was significantly smaller in the CDH non-survivors compared with the findings for the CDH survivors. LVGLS, LVGCS, and RVLS tended to be worse in the CDH non-survivors compared with those in the CDH survivors, but the differences did not reach statistical significance.

### Predicting mortality using the echocardiographic indices

Table 3 summarizes the receiver operating characteristic curve analysis, which we used to predict mortality using the echocardiographic indices. The predictive ability of the univariate logistic regression models was excellent for the LVEDV/RVEDV ratio (specificity: 0.966, sensitivity: 1.00, AUC: 0.98), RVESV/BBW (AUC: 0.96), and RVEDV/BBW (AUC: 0.93), and good for the LVD/RVD ratio in the RV-focused apical four chamber view (AUC: 0.87), LVSV/BBW (AUC: 0.84), LVEDV/BBW (AUC: 0.83), and LV cardiac output (AUC: 0.81) (Table 3).

**Table 3:** Univariate logistic regression analyses of the predictive ability of the echocardiographic indices within 24 h after birth for mortality.

	Threshold	Specificity	Sensitivity	AUC	95% CI
2D echocardiography					
LVDD (M mode, 2D) (mm)	13.3	0.79	0.80	0.78	0.55–1.00
LVDD/BBW (M mode, 2D) (mm)	4.59	0.86	0.83	0.83	0.60–1.00
LVEF (M mode, 2D) (%)	72	0.74	0.75	0.78	0.49–1.00
LAVI (ml/kg)	0.27	0.83	1.00	0.90	0.79–1.00
LVD (RV focused A4CV) (mm)	10.3	0.76	0.67	0.69	0.44–0.94
RVD (RV focused A4CV) (mm)	18.7	0.90	0.67	0.79	0.58–1.00
LVD/RVD (RV focused A4CV)	0.67	0.72	1.00	0.87	0.75–0.99
3D echocardiography					
RVEDV (ml)	5.8	0.62	1.00	0.85	0.70–1.00
RVEDV/BBW (ml/kg)	2.07	0.79	1.00	0.93	0.84–1.00
RVESV (ml)	4.3	0.93	0.83	0.93	0.83–1.00
RVESV/BBW (ml/kg)	1.51	0.90	1.00	0.96	0.90–1.00
RVSV (ml)	3.2	0.90	0.33	0.56	0.27–0.85
RVSV/BBW (ml/kg)	0.83	0.79	0.50	0.56	0.25–0.86
RVEF (%)	27.9	0.93	0.67	0.74	0.46–1.00
RVCO (ml/kg/min)	88.5	0.45	0.67	0.37	0.66–0.97
RVLS (FW) (%)	−13.4	0.72	0.67	0.69	0.45–0.94
RVLS (S) (%)	−24.1	0.35	1.00	0.66	0.44–0.88
LVEDV (ml)	2.9	0.76	1.00	0.83	0.70–0.97
LVEDV/BBW (ml/kg)	1.07	0.72	1.00	0.83	0.69–0.97
LVESV (ml)	1.5	0.72	0.83	0.74	0.59–0.90
LVESV/BBW (ml/kg)	0.595	0.59	1.00	0.72	0.55–0.90
LVSV (ml)	1.2	0.72	1.00	0.84	0.71–0.97
LVSV/BBW (ml/kg)	0.476	0.72	1.00	0.83	0.69–0.96
LVEF (%)	46.5	0.72	1.00	0.79	0.65–0.94
LVCO (ml/kg/min)	54.6	0.79	0.83	0.81	0.65–0.96
LVGCS (%)	−20.0	0.72	1.00	0.83	0.69–0.96
LVGLS (%)	−13.0	0.62	0.83	0.71	0.52–0.91
LV torsion (°/cm)	2.9	0.45	0.83	0.52	0.24–0.80
LVEDV/RVEDV ratio	0.403	0.97	1.00	0.98	0.95–1.00

*AUC* area under the curve, *CI* confidence interval, *LVDD* left ventricular diastolic dimension, *2D* two-dimensional, *3D* three-dimensional, *LVEF* left ventricular ejection fraction, *LAVI* left atrial volume index, *LVD* left ventricular diameter, *RVD* left ventricular diameter, *A4CV* apical four chamber view, *RVEDV* right ventricular end-diastolic volume, *RVESV* right ventricular end-systolic volume, *RVSV* right ventricular stroke volume, *RVEF* right ventricular ejection fraction, *RVCO* right ventricular cardiac output, *RVLS* right ventricular longitudinal strain, *FW* free wall, *S* interventricular septum,*LVEDV* left ventricular end-diastolic volume, *LVESV* left ventricular end-systolic volume, *LVSV* left ventricular stroke volume, *LVCO* left ventricular cardiac output, *LVGCS* left ventricular global circumferential strain, *LVGLS* left ventricular global longitudinal strain, *LV torsion* left ventricular torsion,*BBW* birth body weight.

## Discussion

To the best of our knowledge, this is the first study to show that LV and RV volumes of CDH infants measured by 3D echocardiography with acceptable reproducibility were associated with mortality. We observed that CDH non-survivors had significantly larger RVEDV/BBW and smaller LVEDV/BBW and a markedly smaller LVEDV/RVEDV ratio than those of CDH survivors. The novel index, the LVEDV/RVEDV ratio, was associated with mortality in CDH infants, with the highest AUC of 0.98.

### 3D echocardiography in infants

Infants have much faster heart rates and smaller ventricular sizes compared with adults. In our study, LV and RV volumes were successfully quantified by 3D echocardiography with acceptable reproducibility in CDH infants (Table 2 and Fig. 3). The RV has a complicated shape; hence, RV volume is difficult to quantify by conventional two-dimensional echocardiography. Current 3D analysis is semi-automatic and faithfully traced the RV or LV endocardial borders even in the CDH groups. Furthermore, 3D echocardiography does not use geometric assumptions. Thus, our echocardiographic 3D volume data for CDH infants appears valid.

### Ventricular volumes in CDH patients

Left heart structures are smaller in CDH regardless of the side of the diaphragmatic defect.^33–35^ Primary LV dysfunction in CDH may become apparent owing to the following pathological factors in the transitional period around birth: reduced pulmonary blood flow and LV preload with or without PPHN, LV hypoplasia, smaller LV compressed by a dilated RV,^9^ acute increase in LV afterload at birth, and the negative effects of systemic hypoxia and acidosis.^36^ These factors synergistically reduce LV output and in turn, systemic venous return, resulting in the mildly smaller RV volume in CDH. Fetal rats with CDH induced by administering bis-diamine on the 9^th^ and 10^th^ days of gestation showed cardiac volume reduction involving all four chambers.^34^ RV volume was significantly smaller, but the extent of the RV volume reduction was less than that of LV volume reduction.^37^ Relative RV enlargement and LV hypoplasia could be related to fetal hemodynamics and developmental abnormalities.^38,39^ Relative redistribution of systemic venous return from the ductus venosus and inferior vena cava toward the RV and less to the LV in the CDH fetus may contribute to an imbalance between the left and right ventricles.^39^

### Comparison between CDH survivors and CDH non-survivors, and predicting mortality

The LVEDV/BBW in the CDH non-survivor group was significantly smaller than that in the CDH survivor group (Table 1). This result is similar to findings in a previous report indicating that LVEDV may be an independent predictor of death in CDH.^33^ Our study showed that the CDH non-survivors had larger RVs than those of the CDH survivors (Table 1). The postnatal increased RV afterload, caused by persistently high pulmonary vascular resistance or loss of placental circulation, makes the compliant RV dilate more severely in CDH non-survivors than that in CDH survivors.

The CDH non-survivors had larger RVs and smaller LVs than those of the CDH survivors; however, we did not observe a significant difference in RVEF and LVEF between CDH survivors and CDH non-survivors (Table 1). Focusing on this imbalance between the RV and LV sizes, we tested the LVEDV/RVEDV ratio as a novel index of CHD severity. There was a marked difference in the LVEDV/RVEDV ratio between the CDH survivors and CDH non-survivors (Fig. 3). The LVEDV/RVEDV ratio was associated with mortality, with the greatest AUC of 0.98 (Table 3). This 3D evaluation provided better prediction of mortality compared with two-dimensional evaluation (AUC of LVD/RVD, 0.87). These results indicate that the LVEDV/RVEDV ratio may be a useful index to predict mortality in CDH infants, reflecting the overall quality of the respiratory and circulatory systems caused by organ malformation during the fetal period in these patients.

RV dysfunction and dilation lead to secondary dysfunction in the LV via ventricular interdependence, including shared muscle fibers, pericardial space, and septum.^40,41^ In addition to these mechanical constraints, less RV output reduces pulmonary venous return and, therefore, LV preload. Taken together, LV filling, diastolic function, and output are impaired in cases with RV dysfunction and dilation,^42,43^ as we observed in the CDH group, especially in the CDH non-survivors. The LVEDV/RVEDV ratio using 3D echocardiography can quantitatively reflect ventricular interdependence in CDH patients.

The effect of postnatal responses to afterload on these ventricular volume characteristics needs to be considered. Some of the loading conditions may be affected by higher respiratory support via cardiorespiratory interactions or higher doses of inotropes, especially in the non-survivor group. Afterload is quantified by effective arterial elastance (Ea). Normally, in a given individual, ventricular volume increases in response to an increase in afterload. However, in our CDH patients, RVEDV did not increase as RV Ea increased, and LVEDV and the LVEDV/RVEDV ratio decreased as LV Ea increased (Supplementary Fig. 2). Thus, we do not believe that the ventricular volume characteristics observed in this study were the result of a response to postnatal afterload.

Although there was an association between the LVEDV/RVEDV ratio and respiratory indices, such as o/e LHR or OI (data not shown), cardiac and respiratory status are not always similarly impaired. The data in this study indicated that the LVEDV/RVEDV ratio is strongly associated with mortality in CDH patients although CDH has been believed to be mainly a respiratory disease. Because pulmonary vessels and parenchyma, and LV size, grow in a developmentally influenced manner, we believe that CDH is a cardiorespiratory disorder. Because postnatal evaluation of pulmonary hypoplasia is difficult during intensive care, 3D echocardiography may provide a useful severity assessment that is related to prognosis in CDH infants.

### Study limitations

This is the first study to quantitatively demonstrate, using 3D echocardiography, that the imbalance between LV and RV volumes (the LVEDV/RVEDV ratio) may be useful in assessing CDH severity. However, this study had noteworthy limitations. First, because CDH is a relatively rare disease, the limited sample size in this pilot, single-center study precluded adequate multivariate regression analysis to assess the independence of our main findings from other factors. CDH non-survivors had multiple known risk factors such as lung hypoplasia. Furthermore, ventricular volume is influenced by both intrinsic ventricular stiffness and loading condition, which are in turn influenced by mechanical ventilation, mean airway pressure, drugs, and fluid intake. Multivariate regression analysis is needed to determine whether the 3D echocardiographic data would independently predict the mortality in CDH patients in a future study involving larger numbers of patients. In adults, > 20–25 frames per second is recommended for RV volume measurements.^44^ The frame rate of >40 frames per second that we used in this study, given the fast heart rate in neonates, may not be ideal, but it is also not low, and the evaluation at this frame rate clearly showed the differences between the groups. With six-beat capture, there may be stitching artifacts with the changes in the loading conditions from the first to the 6th beat. However, the included patients were deeply sedated, and body movements were insignificant. Thus, the effect of the stitching artifacts on the results in this study should be minimal.

One may argue that the present results may not be applicable in countries other than Japan owing to differences in the respiratory management strategy. CMV and HFO are the two main ventilatory modes for CDH infants. As an initial ventilatory mode in CDH infants, CMV is often used in Europe and the United States,^45,46^ while HFO is more likely in Japan.^47,48^ How the differences between CMV and HFO affect biventricular volume in CDH patients must be carefully considered. However, this single-center study, performed using a uniform respiratory management policy, showed a marked difference in the LVEDV/RVEDV ratio in CDH survivors and CDH non-survivors. In addition, decreased venous return induced by HFO would affect the LVEDV/RVEDV ratio minimally because a decrease in RV output in turn causes lower LV filling. As another limitation, the single experienced echocardiographer (K Toyoshima) was not blinded to the clinical information of the included patients at the initial 3D data extraction. However, the data extraction processes were semi-automatic, and the intra- and inter-observer variability were acceptable.

In conclusion, our 3D echocardiographic data indicated that the volume difference between the RV and LV was remarkable in severe cases of CDH. Thus, the LVEDV/RVEDV ratio may be a useful parameter to reflect CDH severity. Further prospective multicenter studies involving larger numbers of CDH patients are needed to validate the independent clinical usefulness of the 3D LVEDV/RVEDV ratio over traditional echocardiographic parameters.

## Supplementary information

Supplementary Fig. 1

Supplementary Fig. 2

Supplementary Figure Legends

## Data Availability

The data that support the findings of this study are available from the corresponding author (K Toyoshima) upon reasonable request.
