# Supplementary figures and images for: Right to left ventricular volume ratio is associated with mortality in congenital diaphragmatic hernia
Source: Pediatr Res. 2023 Jan 9;94(1):304–12. doi: 10.1038/s41390-022-02430-z (PMC10356601; doi:10.1038/s41390-022-02430-z)

Supplementary Fig. 1

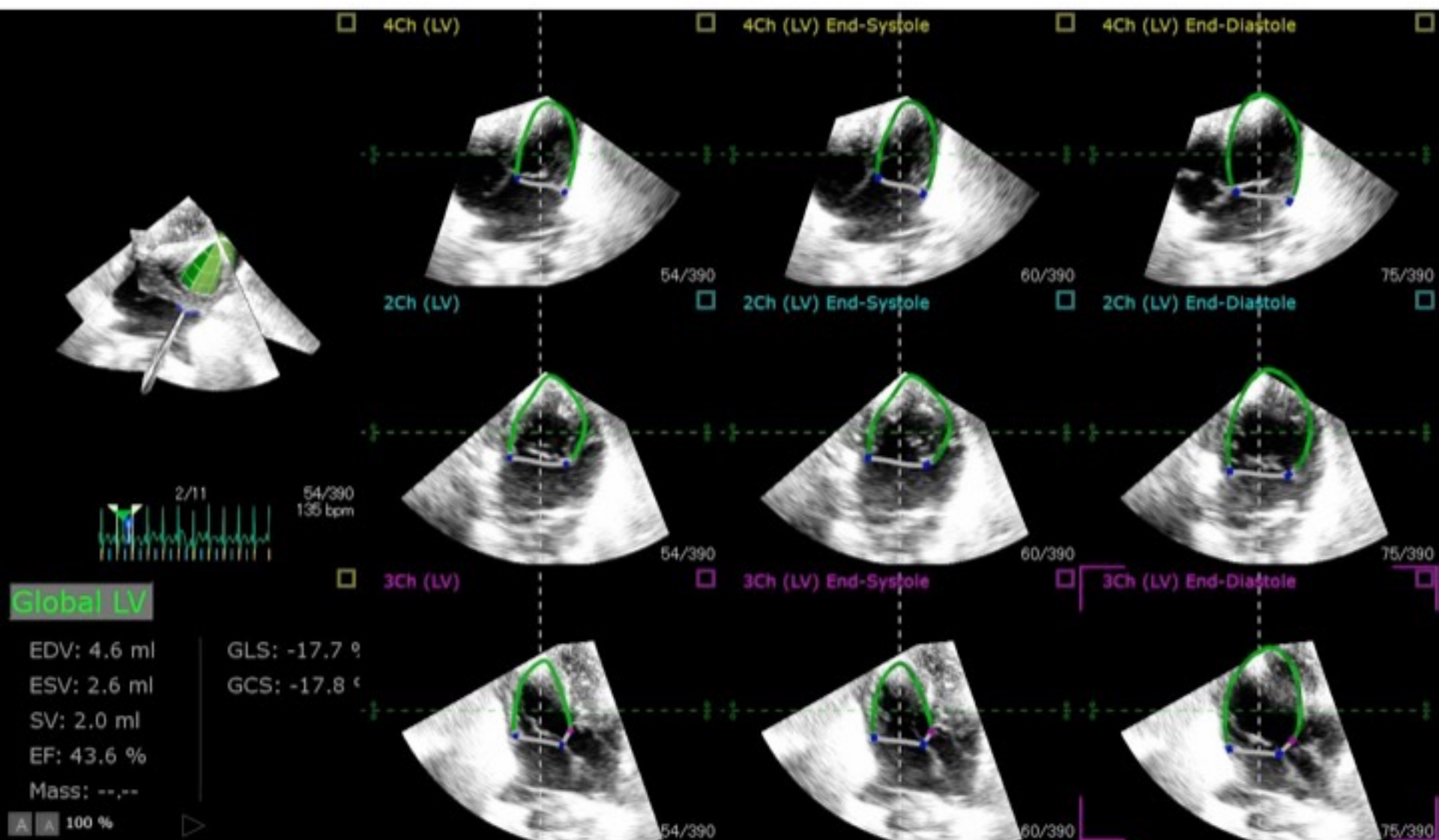

Supplement: Supplementary file 1 — Supplementary Fig. 1 [file 41390_2022_2430_MOESM1_ESM.pdf]

Supplementary Fig. 2

(a)

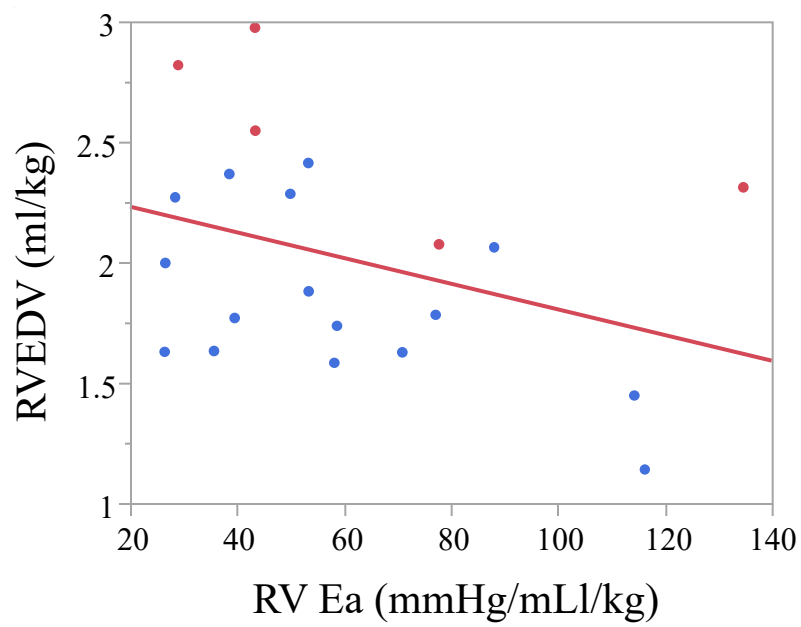

(b)

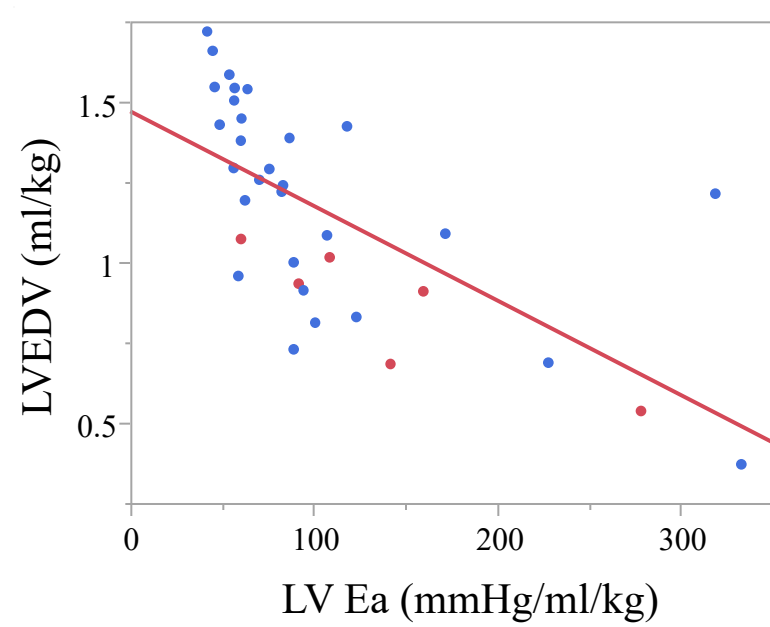

(c)

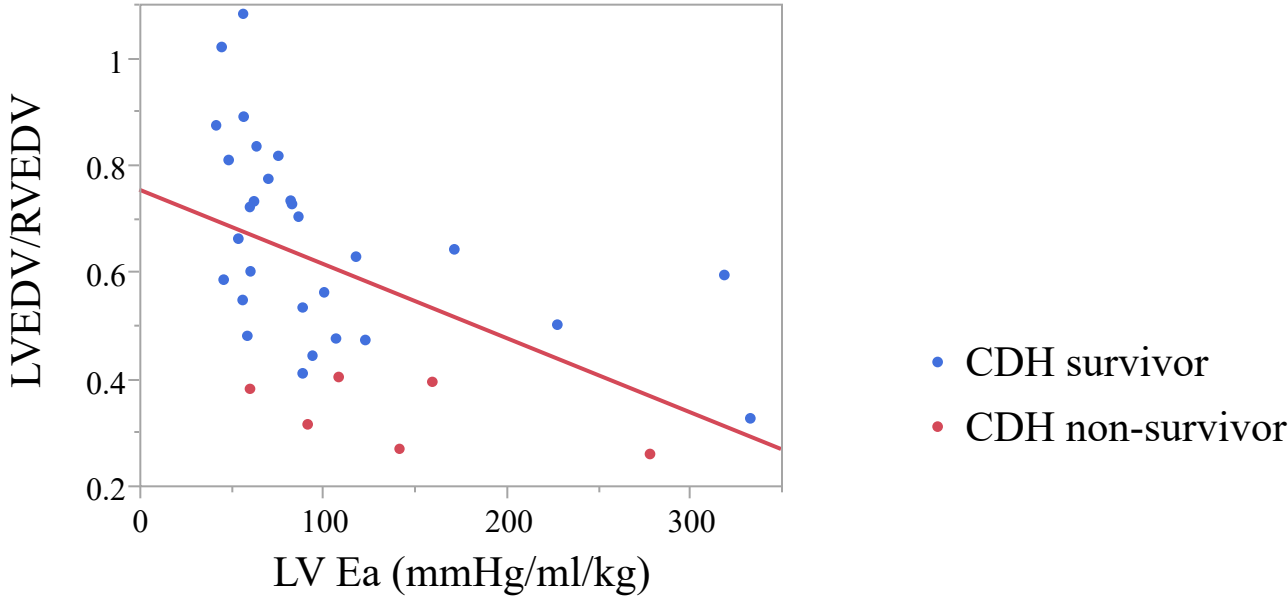

Supplement: Supplementary file 2 — Supplementary Fig. 2 [file 41390_2022_2430_MOESM2_ESM.pdf]
