# Supplementary material for: Right to left ventricular volume ratio is associated with mortality in congenital diaphragmatic hernia
Source: Pediatr Res. 2023 Jan 9;94(1):304–12. doi: 10.1038/s41390-022-02430-z (PMC10356601; doi:10.1038/s41390-022-02430-z)
Supplement: Supplementary file 3 — Supplementary Figure Legends [file 41390_2022_2430_MOESM3_ESM.pdf]

Supplementary Figure Legends

SupplementaryFigure 1. Offline analysis of three-dimensional echocardiographic LV volume calculations at end-diastole. The green outlines indicate the semi-automatically detected LV internal border.

LV left ventricle, *EDV* end-diastolic volume, *ESV* end-systolic volume, *SV* stroke volume, *EF* ejection fraction, *GLS* global longitudinal strain, *GCS* global circumferential strain,*4Ch* four chamber, *3Ch* three chamber *2Ch* two chamber.

Supplementary Figure 2. Effect of afterload on ventricular volume in CDH.

Afterload is quantified by Ea. LV Ea is approximated by the mean blood pressure divided by the LV SV per body weight, and RV Ea is approximated by the TRPG (for those in whom this can be quantified) divided by the RV SV per body weight. (a) RVEDV does not increase as RV Ea increases ($p = 0.109$). (b) LVEDV ($p < 0.0001$) and (c) the LVEDV/RVEDV ratio ($p = 0.002$) decrease as LV Ea increases.

CDH congenital diaphragmatic hernia*, Ea* effective arterial elastance, *SV* stroke volume, *LV* left ventricle, *RV* right ventricle, *TRPG* tricuspid regurgitation pressure gradient, *LVEDV* left ventricular end-diastolic volume, *RVEDV* right ventricular end-diastolic volume.
